# mTOR inhibitor reduces nontumour-related death in liver transplantation for hepatocellular carcinoma

**DOI:** 10.1186/s43556-024-00170-6

**Published:** 2024-03-10

**Authors:** Lincheng Zhang, Peng Liu, Li Zhuang, Sunbin Ling, Qifan Zhan, Wei Zhou, Renyi Su, Lu Yin, Qingyang Que, Jiachen Hong, Jiaqi Bao, Chuxiao Shao, Jinzhen Cai, Shusen Zheng, Xiao Xu

**Affiliations:** 1 Affiliated Hangzhou First People’s Hospital, Zhejiang University School of Medicine, Hangzhou, 310058 China; 2 Key Laboratory of Integrated Oncology and Intelligent Medicine of Zhejiang Province, Hangzhou, 310006 China; 3 Department of Hepatobiliary and Pancreatic Surgery, Lishui Municipal Central Hospital, Lishui, 323000 China; 4 Organ Transplantation Center, The Affiliated Hospital of Qingdao University, Qingdao, 266100 China; 5 Institute of Organ Donation and Transplantation, Department of Medicine, Qingdao University, Qingdao, 266100 China; 6 Shulan (Hangzhou) Hospital, Zhejiang Shuren University School of Medicine, Hangzhou, 310022 China; 7 Department of Hepatobiliary and Pancreatic Surgery, Affiliated Hangzhou First People’s Hospital, Zhejiang University School of Medicine, Hangzhou, 310006 China; 8 Hangzhou Normal University, Hangzhou, 311121 China; 9 Zhejiang Chinese Medical University, Hangzhou, 310058 China; 10 Department of Hepatobiliary and Pancreatic Surgery, Lishui People’s Hospital, Lishui, 323000 China; 11 Department of Hepatobiliary and Pancreatic Surgery, First Affiliated Hospital, Zhejiang University School of Medicine, Hangzhou, 310003 China; 12 National Center for Healthcare Quality Management in Liver Transplant, Hangzhou, 310003 China

**Keywords:** Sirolimus, Liver transplantation, Hepatocellular carcinoma, Nontumour-related death

## Abstract

Sirolimus is a regularly applied immunosuppressant for patients undergoing liver transplantation (LT) for hepatocellular carcinoma (HCC). Sirolimus not only significantly inhibits HCC recurrence but also protects renal function. However, the improvement effect of sirolimus on nontumour-related death in patients is still unknown. The aim of our study was to investigate the therapeutic effect of sirolimus on nontumour-related deaths. In this study, we retrospectively enrolled 403 LT patients with HCC from January 1, 2015, to December 31, 2018. The median follow-up time was 47.1 months. The patients were divided into the sirolimus group (*N* = 184) and the sirolimus-free group (*N* = 219). There were no significant differences between the sirolimus group and the sirolimus-free group in survival (*P* = 0.054). In transplant patients who exceeded the Milan or Hangzhou criteria, the sirolimus group achieved higher survival than the sirolimus-free group (*P* = 0.005; *P* = 0.02). Moreover, multivariate analysis showed that sirolimus strongly reduced the hazard ratio (HR) for nontumour-related death in LT patients who exceeded the Milan (HR: 0.42; 95% CI: 0.18–1; *P* = 0.05) or Hangzhou criteria (HR: 0.26; 95% CI: 0.08–0.89; *P* = 0.032). HCC recurrence increased the risk of nontumour-related death. In conclusion, sirolimus-based immunosuppression can significantly reduce nontumour-related death in LT patients who exceed the criteria for transplantation. In addition, this finding will further promote the application of sirolimus after liver transplantation for hepatocellular carcinoma.

**Supplementary Information:**

The online version contains supplementary material available at 10.1186/s43556-024-00170-6.

## Introduction

Liver failure caused by hepatitis B virus (HBV) and hepatitis C virus (HCV) infection has been the main indication for orthotopic liver transplantation (OLT) worldwide. Because the incidence of hepatocellular carcinoma (HCC) in this patient population is also particularly high, HCC represents an additional indication for OLT in some patients [1–3]. According to the data of the Chinese Liver Transplant Registry from 2015 to 2020, among all indications for LT, liver malignant tumours and liver failure can account for 44.99% and 49.8%, respectively. Due to the high incidence of HBV-related cirrhosis and HCC, the main indications for LT in China are HBV-related liver failure and malignant tumours [4]. After LT, patients are prone to various complications, such as hyperlipemia, diabetes, bleeding, renal insufficiency, cardiovascular complications, and neurological complications. These complications often threaten patient prognosis. Different transplant recipients tend to have different complications. For example, transplant recipients with liver dysfunction are prone to bleeding and thrombosis after transplantation.

Whether the Milan criteria or the expanded criteria proposed by our centre are used, the 5-year overall survival rate of LT for HCC can reach approximately 70% [5, 6]. These LT criteria for HCC mainly consider tumour-related factors, such as tumour morphological characteristics and alpha-fetoprotein (AFP) levels. Therefore, the function of these predictive models is biased towards predicting HCC-related death and recurrence-free survival but does not address nontumour-related death well. A study found that fewer than 1/4 of LT patient deaths were caused by HCC recurrence, while 26.6% of LT recipient deaths were due to nontumour-related reasons, including cardiovascular disease and infection, among 6502 LT patients with HCC [7]. This suggested that death due to causes other than malignant tumours accounted for a large proportion of the deaths in LT patients. Therefore, reducing these deaths is necessary to further improve the prognosis of LT patients. However, very limited studies have explored the reasons behind non-tumour-related deaths in LT for HCC.

Goldberg et al. found that 11 variables were related to nontumour-related death in LT for HCC, such as chronic kidney disease (CKD), INR, etc. [7]. To avoid immune rejection after transplantation, immunosuppressive agents are essential. However, overimmunosuppression can also cause many complications, such as CKD (one-third of long-term LT recipients), new malignant tumours and immunosuppressive-related cardiovascular diseases (i.e., diabetes [14%-61%]). These complications seriously affect the prognosis of patients [8]. In recent years, the proportion of mTOR inhibitors in immunosuppressive agents after liver transplantation for HCC has been increasing. Many studies have revealed that mTOR inhibitors can increase estimated glomerular filtration rate and reduce serum creatinine, renal segmental arterial resistance index and the incidence of proteinuria [9–12]. In addition, a prospective study found that the risk of major cardiac events after LT increased with the deterioration of renal function, and that mTOR inhibitors combined with tacrolimus withdrawal or a small reduction in tacrolimus could improve renal function and the risk of major cardiac events [13].

In view of this, sirolimus may reduce non-HCC recurrence-related deaths, such as renal failure and cardiovascular events. Therefore, we conducted a multi-center retrospective clinical trial to explore the effect of sirolimus on non-HCC recurrent death in LT recipients with HCC in three transplant centres in China.

## Results

### Baseline characteristics of the study groups

**Fig. 1:**
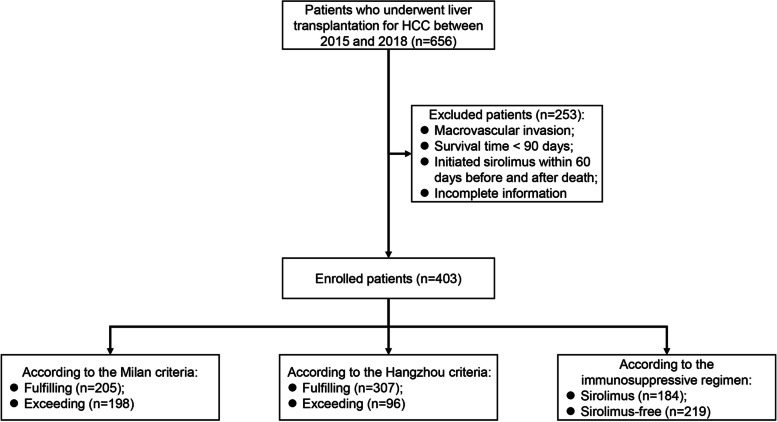
Flowchart for screening patients

A total of 403 patients were eligible for clinical trial inclusion (Fig. 1). Among these patients, 147 patients (80.3%) died of HCC recurrence, and 36 patients (19.7%) died of other causes. The latter included 4 patients with sepsis, 11 patients with multiple organ failure, 6 patients with cerebral hemorrhage, 10 patients with liver failure, 1 patient with biliary complication and 4 patients with unregistered causes. The median follow-up time for the 403 patients was 47.1 months (interquartile range, 17.9—59.4 months). According to the immunosuppressive regimen, they were classified as the sirolimus group (*N* = 184) and the sirolimus-free group (*N* = 219). Table 1 compares the demographic characteristics, biochemical parameters, and tumour pathology characteristics of the two groups.

**Table 1:** Patient- and tumour-specific parameters in patients undergoing liver transplantation for hepatocellular carcinoma grouped by sirolimus-based immunosuppressive agents and sirolimus-free immunosuppressive agents

Characteristics	Control (*n* = 219)	Sirolimus (*n* = 184)	*P*
Recurrence, n (%)			0.455
Yes	67 (30.6)	63 (34.2)	
No	152 (69.4)	121 (65.8)	
Gender, n (%)			0.341
Female	21 (9.6)	24 (13.0)	
Male	198 (90.4)	163 (87.0)	
Age (year), median (IQR)	54.0 [49.0, 59.0]	53.0 [45.0, 59.0]	0.075
BMI, median (IQR)	23.0 [21.5, 24.9]	23.3 [21.0, 25.4]	0.657
Milan criteria, n (%)			0.689
Within	109 (49.8)	96 (52.2)	
Beyond	110 (50.2)	88 (47.8)	
Hangzhou criteria, n (%)			0.907
Within	166 (75.8)	141 (76.6)	
Beyond	53 (24.2)	43 (23.4)	
Capsule invasion, n (%)			0.686
Yes	122 (55.7)	107 (58.2)	
No	97 (44.3)	77 (41.8)	
TNM stage, n (%)			0.49
I	79 (36.1)	55 (29.9)	
II	90 (41.1)	81 (44.0)	
III	47 (21.5)	43 (23.4)	
IV	3 (1.4)	5 (2.7)	
Preoperative RFA, n (%)			0.386
Yes	33 (15.1)	22 (12.0)	
No	186 (84.9)	162 (88.0)	
Preoperative TACE, n (%)			0.82
Yes	77 (35.2)	71 (38.6)	
No	142 (64.8)	113 (61.4)	
Preoperative PEI, n (%)			0.534
Yes	4 (0.02)	2 (0.01)	
No	215 (98.2)	182 (98.9)	
Creatinine (umol/L), median (IQR)	89.0 [64.5, 121.0]	87.5 [62.0, 150.0]	0.721
Albumin (g/L), median (IQR)	35.0 [32.0, 39.8]	36.6 [32.1, 40.4]	0.109
Total bilirubin (umol/L), median (IQR)	126.2 [30.9, 390.8]	100.0 [28.1, 368.0]	0.659
AFP (ng/mL), median (IQR)	23.6 [4.3, 286.4]	36.9 [7.3, 557.0]	0.128
MELD score, median (IQR)	20.0 [11.0, 33.0]	19.0 [10.0, 35.0]	0.286
Child–Pugh score, median (IQR)	9.0 [7.0, 10.0]	9.0 [7.0, 10.0]	0.082
Ascites, n (%)			0.875
without	126 (57.5)	108 (58.7)	
slight	85 (38.8)	68 (37.0)	
serious	8 (3.7)	8 (4.3)	
HE, n (%)			1
without	211 (96.3)	178 (96.7)	
I-II	7 (3.2)	6 (3.3)	
III-IV	1 (0.5)	0 (0.0)	
Bilirubin grade, n (%)			0.252
< 34 umol/L	65 (29.7)	66 (35.9)	
34–50 umol/L	16 (7.3)	17 (9.2)	
> 50 umol/L	138 (63.0)	101 (54.9)	
HBsAg, n (%)			1
Yes	191 (87.2)	160 (87.0)	
No	28 (12.8)	24 (13.0)	
Tumor nodules, n (%)			0.195
1	112 (51.1)	82 (44.6)	
≥ 2	107 (48.9)	102 (55.4)	
Maximal tumor diameter, n (%)			0.251
< 5	72 (32.9)	71 (38.6)	
≥ 5	147 (67.1)	113 (61.4)	

*IQR* Interquartile range, *RFA* Radiofrequency ablation, *TACE* Transcatheter arterial chemoembolization, *PEI* Percutaneous ethanol injection, *MELD* Model for end-stage liver disease, *HE* Hepatic encephalopathy, *PT* Prothrombin time, *BMI* Body mass index

### Analysing factors associated with nontumour-related death in 403 patients

**Fig. 2:**
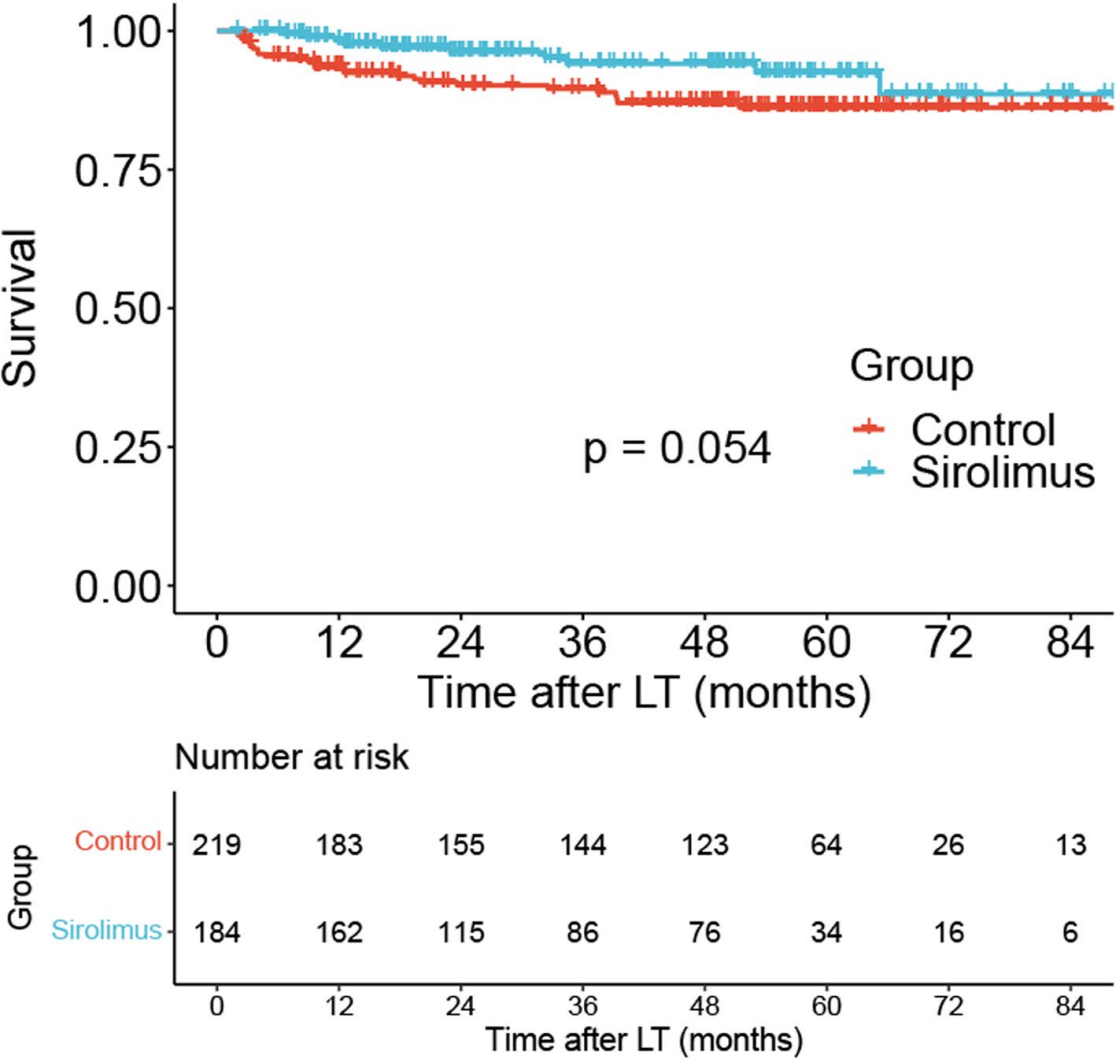
Comparison of survival between the sirolimus group (*N* = 184) and sirolimus-free group (*N* = 219) among all patients (*P* = 0.054)

Multivariate COX regression analysis showed that HCC recurrence (HR: 5.01; 95% CI: 2.48–10.11; *P* < 0.001) was correlated with worse survival in all patients (Table 2). Kaplan‒Meier survival curves of all patients are shown in Fig. 2. There were no significant differences in survival between the sirolimus group (*N* = 184) and sirolimus-free group (*N* = 219) (*P* = 0.054). Ten patients died after LT in the sirolimus group, and 26 patients died after LT in the sirolimus-free group.

**Table 2:** Univariate and multivariate Cox regression analyses of risk factors for survival in all patients

Characteristics	Univariate Cox analysis			Multivariate Cox analysis		
	Hazard Ratio	95% CI	*P*	Hazard Ratio	95% CI	*P*
AFP	1	1–1	0.775			
Age	1	0.96–1.03	0.857			
Gender	1.26	0.49–3.25	0.628			
BMI	0.99	0.9–1.1	0.901			
Creatinine	1.00	1–1.01	0.020	1.00	1–1.01	0.570
MELD score	1.03	1–1.06	0.038	1.02	0.98–1.06	0.252
Sirolimus	0.49	0.24–1.03	0.059			
Recurrence	5.19	2.58–10.44	< 0.001	5.01	2.48–10.11	< 0.001

*AFP* Alpha fetoprotein, *BMI* Body mass index, *MELD* Model for end-stage liver disease

### Subgroup analysis of the benefits of sirolimus based on transplantation criteria

**Fig. 3:**
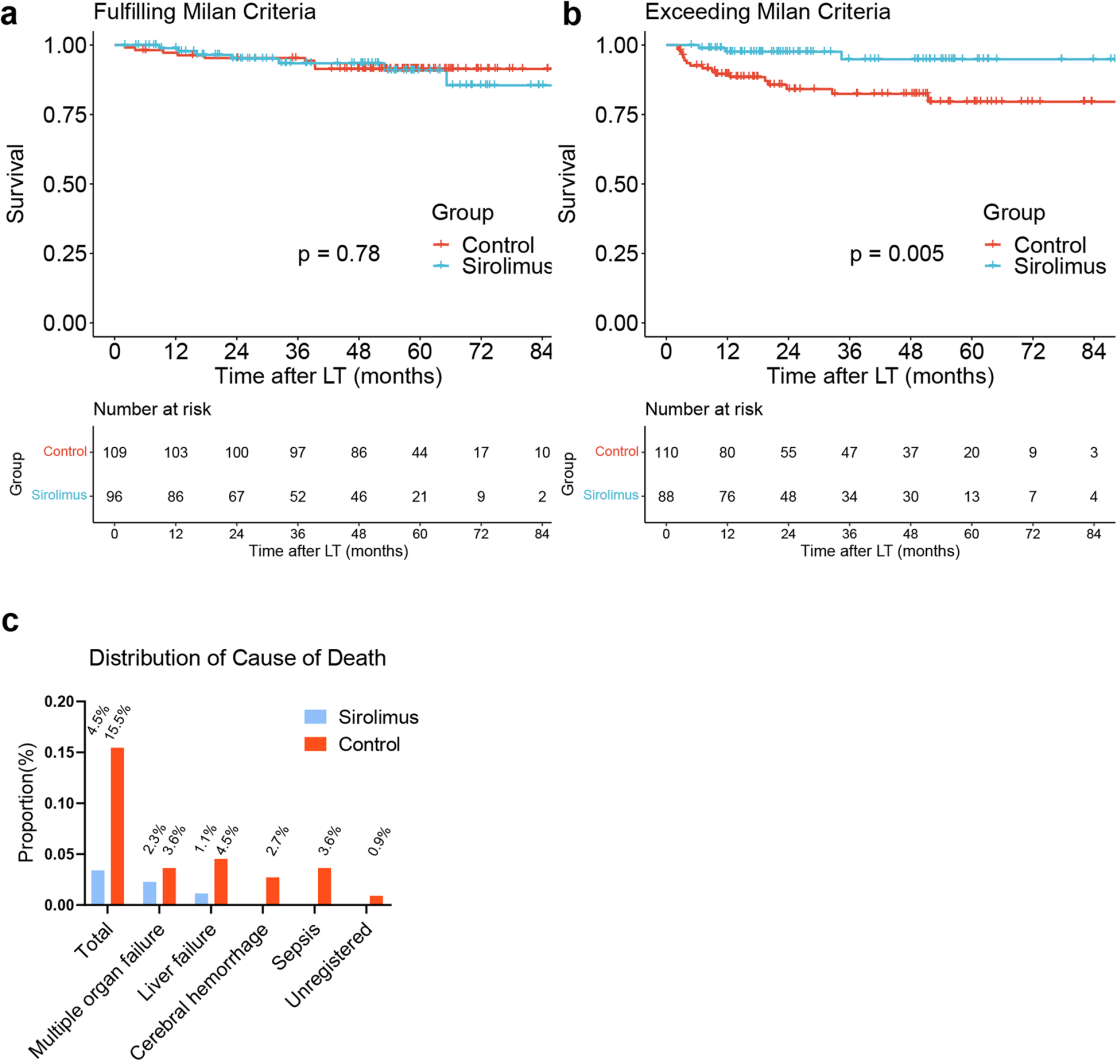
Comparison of survival between the sirolimus group (*N* = 96) and sirolimus-free group (*N* = 109) among patients fulfilling the Milan criteria (*P* = 0.78) (**a**). Comparison of survival between the sirolimus group (*N* = 88) and sirolimus-free group (*N* = 110) among patients exceeding the Milan criteria (*P* = 0.005) (**b**). The distribution of causes of death in patients exceeding the Milan criteria was distinguished by sirolimus administration (**c**)

Among LT patients with HCC who met the Milan criteria (*N* = 205), there was no significant difference (*P* = 0.78) in survival between the sirolimus group (*N* = 96) and the sirolimus-free group (*N* = 109) (Fig. 3a). Conversely, in LT patients with HCC who exceeded the Milan criteria (*N* = 198), sirolimus (*N* = 88) significantly improved survival compared with no sirolimus treatment (*N* = 110) (*P* = 0.005) (Fig. 3b). Sirolimus treatment (HR: 0.22; 95% CI: 0.06–0.76; *P* = 0.016), model for end-stage liver disease (MELD) score (HR: 1.06; 95% CI: 1.01–1.1; *P* = 0.011) and HCC recurrence (HR: 2.69; 95% CI: 1.07–6.74; *P* = 0.035) were independent prognostic factors for survival (Table 3). The histogram depicts the proportion of nontumor-related causes of death in the two groups (Fig. 3c). Specifically, sirolimus reduced deaths due to sepsis (0% vs. 3.6%), multiple organ failure (2.3% vs. 3.6%), liver failure (1.1% vs. 4.5%), cerebral hemorrhage (0% vs. 2.7%) and unregistered causes (0 vs. 0.9%).

**Table 3:** Univariate and multivariate Cox regression analyses of risk factors for survival in patients exceeding Milan criteria

Characteristics	Univariate Cox analysis			Multivariate Cox analysis		
	Hazard Ratio	95%CI	*P*	Hazard Ratio	95%CI	*P*
AFP	1	1–1	0.712			
Age	1.03	0.98–1.09	0.202			
Gender	0.94	0.22–4.06	0.936			
BMI	0.93	0.8–1.07	0.311			
Creatinine	1	1–1.01	0.150			
MELD score	1.06	1.01–1.1	0.007	1.06	1.01–1.1	0.011
Sirolimus	0.20	0.06–0.70	0.011	0.22	0.06–0.76	0.016
Recurrence	3.05	1.2–7.77	0.019	2.69	1.07–6.74	0.035

*AFP* Alpha fetoprotein, *BMI* Body mass index, *MELD* Model for end-stage liver disease

**Fig. 4:**
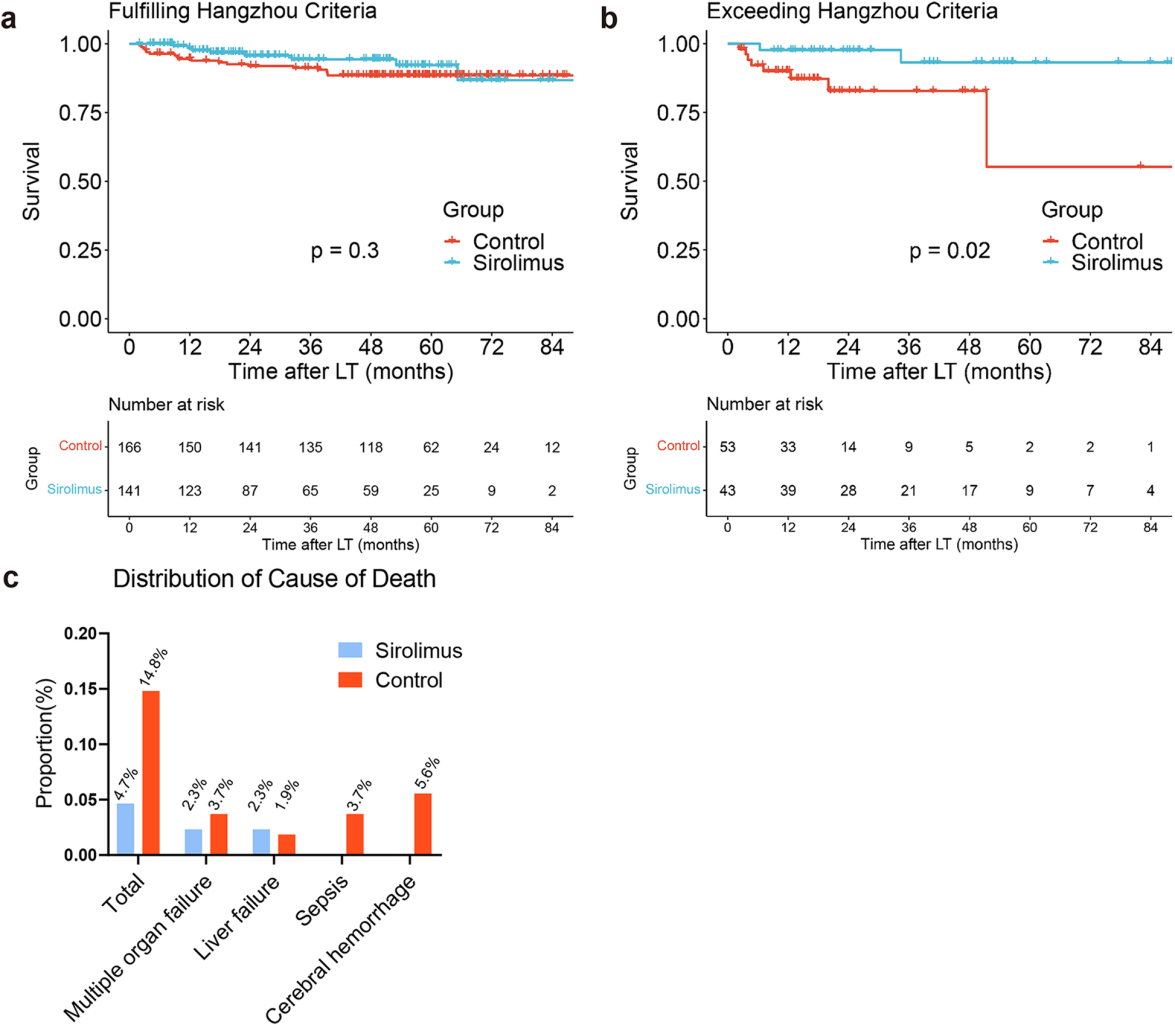
Comparison of survival between the sirolimus group (*N* = 141) and sirolimus-free group (*N* = 166) among patients fulfilling the Hangzhou criteria (*P* = 0.3) (**a**). Comparison of survival between the sirolimus group (*N* = 43) and sirolimus-free group (*N* = 53) among patients exceeding the Hangzhou criteria (*P* = 0.02) (**b**). The distribution of causes of death in patients exceeding the Hangzhou criteria was distinguished by sirolimus administration (**c**)

Among the 307 LT patients with HCC fulfilling the Hangzhou criteria, no significant difference in survival (*P* = 0.3) was found between the sirolimus group (*N* = 141) and the sirolimus-free group (*N* = 166) (Fig. 4a). Conversely, among the 96 LT patients with HCC exceeding the Hangzhou criteria, sirolimus (*N* = 43) significantly improved survival (N = 53) (*P* = 0.02) (Fig. 4b). Supplementary Table 1 shows that sirolimus treatment (HR: 0.27; 95% CI: 0.05–1.46; *P* = 0.129) was not an independent prognostic factor for survival. The histogram depicts the proportion of nontumor-related causes of death in the two groups (Fig. 4c). Specifically, sirolimus reduced deaths due to sepsis (0% vs. 3.7%), multiple organ failure (2.3% vs. 3.7%) and cerebral hemorrhage (0% vs. 5.6%).

### Effect of hepatocellular carcinoma recurrence on nontumour-related death

**Fig. 5:**
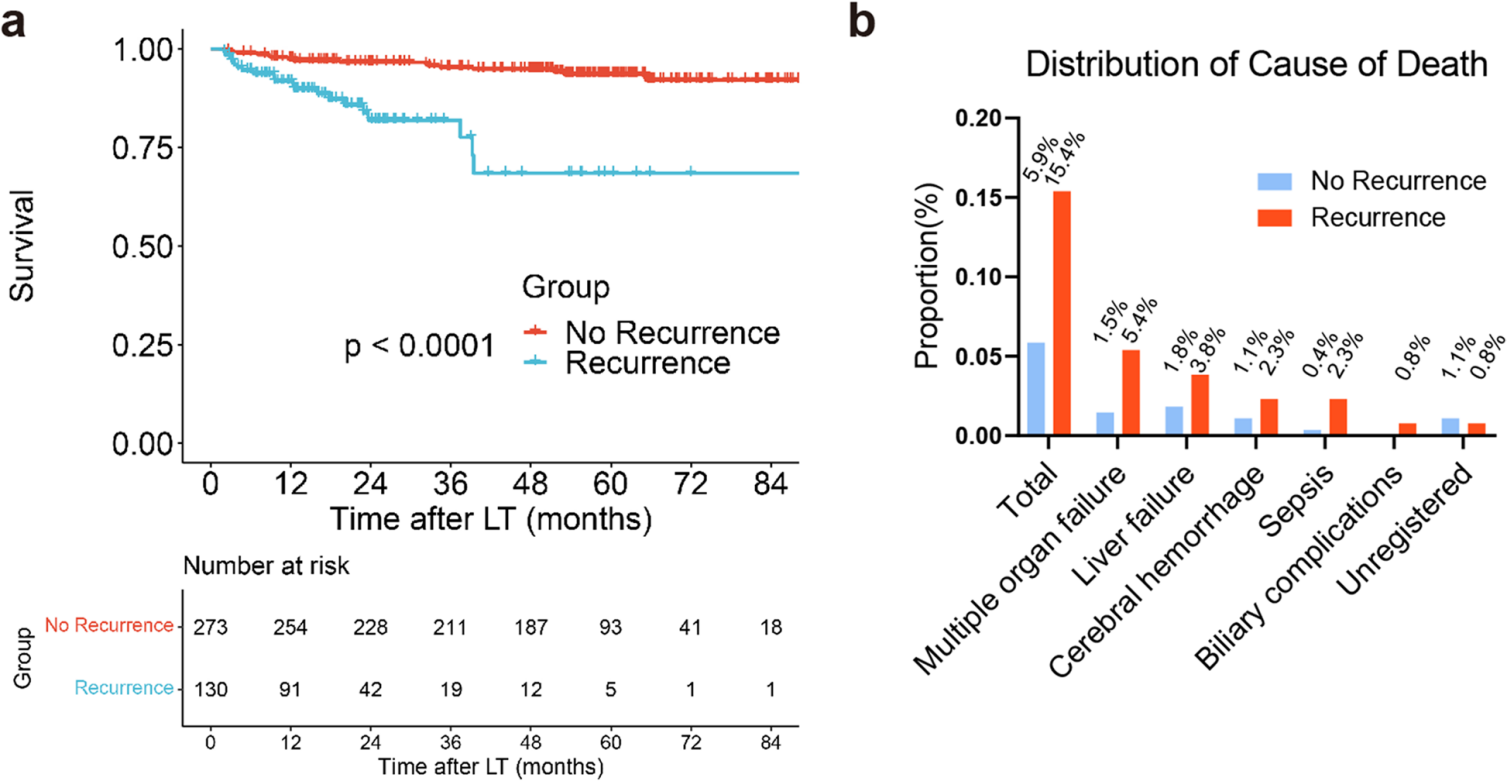
Comparison of survival between patients with (*N* = 130) and without hepatocellular carcinoma recurrence (*N* = 273) (*P* < 0.0001) (**a**). Distribution of causes of death, recurrence vs. nonrecurrence group (**b**)

The recurrence group had higher death rates than the nonrecurrence group (15.4% vs. 5.9%, *P* < 0.001) (Fig. 5a-b). Specifically, more deaths due to sepsis (2.3% vs. 0.4%), multiple organ failure (5.4% vs. 1.5%), cerebral hemorrhage (2.3% vs. 1.1%), liver failure (3.8% vs. 1.8%) and biliary complication (0.8% vs. 0%) occurred in the recurrence group than in the nonrecurrence group (Fig. 5b).

## Discussion

In our cohort, the nontumour-related death rate was 29.5%, which was similar to the 26.6% rate reported by Goldberg et al. [7]. The main aetiology of liver disease in this cohort was HBV, while the most common aetiologies of liver disease in the OPTN/UNOS database were HCV and nonalcoholic steatohepatitis. Therefore, the difference in aetiologies may not be the main factor affecting nontumour-related death.

In our study, liver dysfunction increases the risk of nontumour-related death in patients who beyond the Milan criteria. The liver is an important organ involved in many physiological processes, such as energy metabolism, immune system support, and substance synthesis and decomposition [14]. Patients with poor liver function may have low immunity and are prone to various complications affecting prognosis. However, for patients exceeding the Hangzhou criteria, liver function did not affect nontumour-related death. This suggests that liver function only affects people with a relatively good prognosis. Moreover, one of the indications for liver transplantation is poor liver function. Therefore, liver function may not be a very important factor in nontumour-related death.

Interestingly, we found that transplant patients with HCC recurrence were more likely to experience nontumour-related death, but the underlying mechanism is not clear. This may be attributed to the side effects of antitumour drugs or treatments taken after HCC recurrence. The HCC recurrence rate is higher in patients who exceed the criteria for LT than in those who fulfil the criteria for LT [15]. Therefore, for patients exceeding the criteria for LT, the high risk of nontumour-related death may be due to the high HCC recurrence rate.

This study found that sirolimus administration can significantly reduce nontumour-related death in LT patients whose status puts them beyond the Milan or Hangzhou criteria. The activation of mTOR pathway is closely related to the proliferation of HCC, tumor metabolic reprogramming and tumor angiogenesis [16, 17]. As an mTOR inhibitor, sirolimus may decrease nontumour-related death by reducing the HCC recurrence rate in patients exceeding the transplant criteria [12, 18]. Importantly, no deaths due to sepsis occurred in the sirolimus group among those who were beyond the Milan or Hangzhou criteria. Similarly, compared with the standard exposure group of calcineurin inhibitors (CNIs), the infection rate of cytomegalovirus and BK virus was lower in renal transplant recipients treated with everolimus combined with CNIs reduction [19, 20]. In addition, compared with placebo, patients with lupus nephritis have a higher incidence of pneumonia after CNIs treatment [21]. Therefore, there may be a direct correlation between CNI exposure and the reduction of anti-infection ability. Ruiz-García et al. found by whole-exome or targeted sequencing that the PI3K110δ mutation leads to natural killer cell developmental phenotype changes and cytotoxic dysfunction, which in turn causes sepsis. After the administration of sirolimus in these patients, these defects were partially restored [22]. For patients with methicillin-resistant Staphylococcus aureus-induced sepsis, sirolimus downregulates the proportions of Th1 and Th17 cells by activating autophagy to alleviate organ damage [23]. The intestinal barrier has the effect of preventing microbial invasion. Sirolimus can up-regulate the expression of polo-like kinase 1 in intestinal epithelial cells to promote autophagy and inhibit apoptosis, thereby improving intestinal barrier function [24]. Thus, sirolimus may treat and prevent sepsis by bolstering the immune microenvironment and maintaining the intestinal barrier.

Renal insufficiency is common after transplantation because of the side effects of immunosuppressive agents and haemodynamic changes [25, 26]. A type of immunosuppressant, CNIs damage renal function. In contrast, mTOR inhibitors can increase the estimated glomerular filtration rate and reduce serum creatinine, the renal segmental arterial resistance index and the incidence of proteinuria [9, 10, 27]. Renal insufficiency is closely related to the course of infection [28, 29]. Renal insufficiency causes changes in many different substances in the body fluids and urine, such as urea, creatinine and organic acids. These changes affect the expression of virulence factors, as well as the activity of specific immunity [30]. Thus, the protection of renal function by sirolimus can also prevent the occurrence of infection after transplantation to a certain extent. At the same time, sepsis can also promote the occurrence of renal insufficiency, such as sepsis-related acute kidney injury. This event is an important cause of death in critically ill patients [31–35]. In addition, renal insufficiency increases the risk of cardiovascular disease [36–41]. One prospective study found that the risk of major cardiac events after LT increased with the deterioration of renal function and that mTOR inhibitors combined with tacrolimus withdrawal or a small reduction in tacrolimus could improve renal function and the risk of major cardiac events [13].

Likewise, the sirolimus group had no neurological complications in transplant patients beyond the Milan or Hangzhou criteria. As a common vascular abnormality in the central nervous system, cerebral cavernous malformations (CCMs) require the upregulation of the phosphatidylinositol-3-kinase-mTOR pathway and the loss of CCM complex function to grow. Thus, sirolimus can effectively block the formation of CCMs by inhibiting the mTOR pathway in mouse models [42]. Two patients with brain involvement and cognitive impairment in Sturge‒Weber syndrome showed significant improvements in anger, cognitive function and depression after taking sirolimus [43]. Because of the limited number of cases, the therapeutic effect of sirolimus on neurological complications after transplantation is not clear, but its specific mechanism is also worth further exploration.

Another study showed that sirolimus administration at a dose of 2.24 mg per kg of body weight per day from 270 days of age to 600 days of age significantly prolonged the lifespan of mice [44]. Consequently, mTOR is probably important in age-related pathological progression. It is also worth further exploring other biological functions.

This study has some limitations. The cause of death of some transplant patients was unknown. In addition, the clinical baseline information of the donor and the cold ischemia time of the donor liver were not fully considered. In the future, larger multicentre, prospective clinical studies will be needed to clarify how well sirolimus improves nontumour-related death. The target population of this study was only patients who underwent liver transplantation for HCC, but sirolimus is also widely used in liver transplantation without HCC and other organ transplantation. Therefore, whether sirolimus has a protective effect on nontumour-related death in these patients is worth exploring.

In summary, this study first found that sirolimus reduced nontumour-related death in patients receiving LT for HCC. Our summary of the experience of three transplant centres can promote the application of sirolimus as an immunosuppressant after LT for HCC.

## Materials and methods

### Study design

Patients with HCC who underwent LT at three LT centres (The First Affiliated Hospital of Zhejiang University, the Affiliated Hospital of Qingdao University, and Shulan (Hangzhou) Hospital) between January 1, 2015, and December 31, 2018, were retrospectively enrolled. The required information obtained from the patients included patient demographics, comorbidities, laboratory tests, radiological data, tumour pathology, treatment before LT, surgical data and HCC samples. The MELD score is a scoring system for evaluating liver function reserve and prognosis in patients with chronic liver disease based on creatinine, international normalized ratio (INR), and bilirubin combined with cirrhosis etiology. The MELD score was calculated as: MELD = 3.78 × ln [T-BiL (mg/dl)] + 11.2 × ln [INR] + 9.57 × ln [Cr (mg/dl)] + 6.43. The inclusion criteria were as follows: (a) age over 18 years; (b) first-time LT; (c) survival ≥ 90 days after transplantation to ensure that sirolimus has sufficient onset time; and (d) no macrovascular invasion. The exclusion criteria were as follows: (a) the patient underwent multiorgan transplantation; (b) sirolimus was started within 60 days before death; (c) the patient had a pathological diagnosis of non-HCC after transplantation; (d) the clinical data were incomplete; and (e) the patient’s primary tumour was derived from extrahepatic organs.

At the same time, determine whether the included patients meet the Milan criteria or Hangzhou criteria. Milan criteria requires the following conditions: (a) a single tumor diameter does not exceed 5 cm or more multiple tumors less than 3 and the maximum diameter does not exceed 3 cm; (b) no vascular invasion; (c) there were no signs of lymph node or extrahepatic metastasis. Hangzhou criteria requires the following conditions: (a) no vascular invasion and extrahepatic metastasis; (b) the sum of all tumor diameter ≤ 8 cm, or the sum of all tumor nodule diameter > 8 cm, but alpha-fetoprotein (AFP) < 400 ng / ml and histological grade was high and moderate differentiation.

### Immunosuppressive regimen

In all patients, basiliximab (20 mg) was regularly administered within 2 h before surgery and on the fourth day after surgery. Methylprednisolone (5 mg/kg) was intraoperatively administered. An immunosuppressive regimen based on tacrolimus/cyclosporin A + mycophenolate was implemented in the early postoperative period. In the sirolimus group, sirolimus was usually started 30–60 days after transplantation. The blood concentration of sirolimus was stable at 4–10 ng/ml. At the initiation of sirolimus treatment, the CNI dose was reduced to half, and the CNI was discontinued when the sirolimus target level was reached. Tacrolimus/cyclosporin A was continued in the sirolimus-free group, and its dose was adjusted according to liver function and the blood immunosuppressant concentration. Both groups were treated with mycophenolate.

### Primary endpoint

The primary endpoint of our study was survival in the transplant patients.

### Statistical analysis

Continuous data with a normal distribution are expressed as the mean and standard deviation. Nonnormally distributed continuous data are expressed as the median and interquartile range. Categorical data are expressed as numbers (percentages). In the survival analysis, the survival outcome of patients who died due to tumour-related reasons was defined as censored. All statistical tests were two-tailed, with *p* < 0.05 indicating statistical significance. Statistical analysis was performed using R statistical software (version 4.2.0, https://www.r-project.org). The packages “survival”, “Formula”, “ggplot2”, and “readxl” were used.

### Supplementary Information

**Additional file 1: Supplemental Table 1. **Univariate and multivariate Cox regression analyses of risk factors for survival in patients exceeding Hangzhou criteria.

## Data Availability

The raw data of this manuscript is available by the corresponding authors to qualifed researchers upon reasonable request.

## Abbreviations

BMI: Body mass index
CCMs: Cerebral cavernous malformations
CMV: Cytomegalovirus
CNIs: Calcineurin inhibitors
HR: Hazard ratio
HCC: Hepatocellular carcinoma
LT: Liver transplantation
MELD: Model for end-stage liver disease
Mtor: Mammalian target of rapamycin
