# Supplementary material for: mTOR inhibitor reduces nontumour-related death in liver transplantation for hepatocellular carcinoma
Source: Mol Biomed. 2024 Mar 10;5:9. doi: 10.1186/s43556-024-00170-6 (PMC10924815; doi:10.1186/s43556-024-00170-6)
Supplement: Supplementary file 1 — Additional file 1: Supplemental Table 1. Univariate and multivariate Cox regression analyses of risk factors for survival in patients exceeding Hangzhou criteria. [file 43556_2024_170_MOESM1_ESM.docx]

**Title Page**

**mTOR inhibitor reduces nontumour-related death in liver transplantation for hepatocellular carcinoma**

**Authors**

Lincheng Zhang, MD, ^1, 2, 3^, Peng Liu, MD, ^4, 5^, Li Zhuang, MD, ^6^, Sunbin Ling, MD, ^7^, Qifan Zhan, MD, ^1, 2^, Wei Zhou, MD, ^1, 2^, Renyi Su, MD, ^1, 2^, Lu Yin, MD, ^2^, Qingyang Que, MD, ^1, 2^, Jiachen Hong, MD, ^8^, Jiaqi Bao, MD, ^9^, Chuxiao Shao, MD, ^10^, Jinzhen Cai, MD, ^4, 5^, Shusen Zheng, MD, PhD, ^6, 11^, Xiao Xu, MD, PhD, ^1, 2, 12^

**Affiliations**

^1^Zhejiang University School of Medicine, Hangzhou, 310058, China

^2^Key Laboratory of Integrated Oncology and Intelligent Medicine of Zhejiang Province, Hangzhou, 310006, China

^3^Department of Hepatobiliary and Pancreatic Surgery, Lishui Municipal Central Hospital, Lishui, 323000, China

^4^Organ Transplantation Center, The Affiliated Hospital of Qingdao University, Qingdao, 266100, China

^5^Institute of Organ Donation and Transplantation, Department of Medicine, Qingdao University, Qingdao, 266100, China

^6^Shulan (Hangzhou) Hospital, Zhejiang Shuren University School of Medicine, Hangzhou, 310022, China

^7^Department of Hepatobiliary and Pancreatic Surgery, Affiliated Hangzhou First People's Hospital, Zhejiang University School of Medicine, Hangzhou, 310006, China

^8^Hangzhou Normal University, Hangzhou, 311121, China

^9^Zhejiang Chinese Medical University, Hangzhou, 310058, China

^10^Department of Hepatobiliary and Pancreatic Surgery, Lishui People’s Hospital, Lishui, 323000, China

^11^Department of Hepatobiliary and Pancreatic Surgery, First Affiliated Hospital, Zhejiang University School of Medicine, Hangzhou, 310003, China.

^12^National Center for Healthcare Quality Management in Liver Transplant, Hangzhou, 310003, China.

Lincheng Zhang, Peng Liu and Li Zhuang contributed equally to this work.

ORCID number: Lincheng Zhang (0000-0001-6989-1399); Sunbin Ling (0000-0003-0846-5489); Shusen Zheng (0000-0003-1459-8261); Xiao Xu (0000-0002-2761-2811).

**Address for Correspondence**

Jinzhen Cai, Organ Transplantation Center, The Affiliated Hospital of Qingdao University, Qingdao, China

Email: caijinzhen@qdu.edu.cn

Shusen Zheng, Department of Hepatobiliary and Pancreatic Surgery, First Affiliated Hospital, Zhejiang University School of Medicine, Hangzhou, China.

Email: shusenzheng@zju.edu.cn

Xiao Xu, Zhejiang University, School of Medicine, Hangzhou, China.

Email: [zjxu@zju.edu.cn](mailto:zjxu@zju.edu.cn)

**Keywords** Sirolimus, Liver transplantation, Hepatocellular carcinoma, Nontumour-related death.

**Supplemental Table 1** Univariate and multivariate Cox regression analyses of risk factors for survival in patients exceeding Hangzhou criteria.

| Characteristics | Univariate Cox analysis | | | Multivariate Cox analysis | | |
| --- | --- | --- | --- | --- | --- | --- |
|  | Hazard Ratio | 95%CI | P | Hazard Ratio | 95%CI | P |
| AFP | 1 | 1-1 | 0.711 |  |  |  |
| Age | 1.04 | 0.97-1.11 | 0.247 |  |  |  |
| Gender | 1.12 | 0.14-8.88 | 0.913 |  |  |  |
| BMI | 0.88 | 0.7-1.1 | 0.271 |  |  |  |
| Creatinine | 1 | 1-1.01 | 0.409 |  |  |  |
| MELD score | 1.05 | 0.99-1.12 | 0.100 |  |  |  |
| Sirolimus | 0.18 | 0.04-0.89 | 0.035 | 0.27 | 0.05-1.46 | 0.129 |
| Recurrence | 5.25 | 1.19-23.2 | 0.029 | 3.27 | 0.71-15.14 | 0.130 |

Note: AFP, alpha fetoprotein; BMI, body mass index; MELD, model for end-stage liver disease.
